# Food Anthocyanins: Malvidin and Its Glycosides as Promising Antioxidant and Anti-Inflammatory Agents with Potential Health Benefits

**DOI:** 10.3390/nu15133016

**Published:** 2023-07-01

**Authors:** Anna Merecz-Sadowska, Przemysław Sitarek, Tomasz Kowalczyk, Karolina Zajdel, Mariusz Jęcek, Paweł Nowak, Radosław Zajdel

**Affiliations:** 1 Department of Economic and Medical Informatics, University of Lodz, 90-214 Lodz, Poland; mariusz.jecek@uni.lodz.pl (M.J.); pawel.nowak@uni.lodz.pl (P.N.); radoslaw.zajdel@uni.lodz.pl (R.Z.); 2 Department of Medical Biology, Medical University of Lodz, 90-151 Lodz, Poland; przemyslaw.sitarek@umed.lodz.pl; 3 Department of Molecular Biotechnology and Genetics, Faculty of Biology and Environmental Protection, University of Lodz, 90-237 Lodz, Poland; tomasz.kowalczyk@biol.uni.lodz.pl; 4 Department of Medical Informatics and Statistics, Medical University of Lodz, 90-645 Lodz, Poland; karolina.smigiel@umed.lodz.pl

**Keywords:** malvidin, malvidin glycosides, antioxidant activity, anti-inflammatory activity, health benefits

## Abstract

Anthocyanins are flavonoid compounds that are abundantly present in fruits and vegetables. These compounds contribute to the color of these foods and offer various health benefits to consumers due to their biological properties. There are more than 1000 types of anthocyanins in nature, all derived from 27 anthocyanidin aglycones that have different glycosylations and acylations. Malvidin is one of the most well-known anthocyanidins. Several studies, including those conducted on cell lines, animals, and humans, have suggested that malvidin and its glycosides possess anti-carcinogenic, diabetes-control, cardiovascular-disease-prevention, and brain-function-improvement properties. These health benefits are primarily attributed to their antioxidant and anti-inflammatory effects, which are influenced by the molecular mechanisms related to the expression and modulation of critical genes. In this article, we review the available information on the biological activity of malvidin and its glycosides concerning their health-promoting effects.

## 1. Introduction

Flavonoids are a class of polyphenolic compounds found ubiquitously in plants and encompass a diverse range of chemical structures and characteristics. They consist of a 15-carbon atom skeleton (C6-C3-C6) comprising three rings: two phenolic rings (A and B) and one pyran ring (C) [1,2]. Anthocyanins are water-soluble flavonoids that are differentiated based on the nature, number, and location of sugars attached to the molecule; the number of aliphatic or aromatic acids attached to sugars; the number of hydroxyl groups present; and the degree of methylation of hydroxyl groups. Malvidin, peonidin, cyanidin, pelargonidin, petunidin, and delphinidin are some of the most abundant anthocyanin aglycones. Anthocyanins aglycones are glycosidically linked to sugars in the C-3 position of the anthocyanidin. The glycone part of anthocyanins provides chemical stability and solubility, while the conjugated double bonds in the anthocyanidin moiety are responsible for light absorption and unique color production. Generally, methoxylation imparts a red color, while increased hydroxylation produces a blue pigment [3,4].

It has been reported that more than 1000 distinct anthocyanins have been identified to date [5]. These molecules are prevalent in plants and are responsible for the red, purple, and blue pigments observed in a variety of fruits, vegetables, and their derivatives [6]. The composition of anthocyanins may differ based on various factors, such as the source of the food and its variety [7], as well as seasonal changes and environmental conditions [8]. Additionally, factors such as the state of the food, whether fresh, frozen, or dried [9]; and the manner in which the food is stored and prepared, for instance, whether it is peeled or unpeeled, can also play a role in the concentration of anthocyanins [7,10]. The quantity of anthocyanins found in fruits and vegetables can vary significantly, with levels ranging from 30 to 1500 mg/100 g [11]. These compounds exhibit a wide range of biological activities, including antioxidant and anti-inflammatory properties, which are associated with their potential to provide anti-carcinogenic, cardioprotective, antidiabetic, and neuroprotective properties [12]. Moreover, due to their beneficial effects on human health, anthocyanins may be used in food supplements [13].

Malvidin (PubChem CID: 159287), named 3′,5′-dimethoxy-3,4′,5,7-tetrahydroxyflavylium acid anion, is a type of anthocyanidin cation whose chemical structure is similar to delphinidin but has methyl groups attached to positions 3′ and 5′. This compound is present in various fruits, vegetables, and their derivatives, and it accounts for approximately 7% of the pigment distribution in edible plant parts. Malvidin is commonly linked to various sugar moieties in the C-3 position. This molecule has four hydrogen-bond donors, making it a potent scavenger of reactive oxygen species (ROS) [14]. Malvidin and its glycosides play an important role as antioxidant and anti-inflammatory agents [15].

There has been a significant increase in the frequency of articles published on the properties of anthocyanins. However, malvidin, a potent antioxidant and anti-inflammatory agent, has been relatively less studied among anthocyanidins. Therefore, this review article aims to discuss the biological activity and potential health benefits of malvidin and its glycosides.

## 2. Study Design

Published data were explored using databases such as NCBI-PubMed, Google Scholar, Scopus, and ScienceDirect. The following keywords were used: malvidin, malvidin glycosides, in vitro models, in vivo models, antioxidant potential of malvidin and its glycosides, anti-inflammatory potential of malvidin and its glycosides, anticancer potential of malvidin and its glycosides, antidiabetic potential of malvidin and its glycosides, cardioprotective potential of malvidin and its glycoside, and neuroprotective potential of malvidin and its glycosides. This review of the literature encompassed studies investigating the effects of malvidin and its glycosides on cellular systems, animal models, and human models.

## 3. Exploring the Characteristic and Food Sources of Malvidin and Other Anthocyanins

Anthocyanins are a group of water-soluble pigments that can be found in all higher plant tissues, such as leaves, stems, roots, flowers, and fruits. These compounds are produced via the phenylpropanoid pathway, which is part of the plant’s secondary metabolism. Anthocyanins play several important functions that are essential for the plant’s survival. The primary role of anthocyanins is to attract animals and pollinating insects, aiding in the dissemination of seeds or the spread of pollen. Moreover, it has been demonstrated that the synthesis of anthocyanins is induced during the establishment of adverse conditions, suggesting their involvement in both biotic and abiotic stresses [16,17].

Anthocyanins are a class of flavonoid derivatives characterized by a flavylium cation backbone, which is hydroxylated at various positions, typically on carbons C3, C5, C6, C7, C3′, C4′, and C5′. This results in a large class of approximately 1000 different anthocyanin derivatives, each consisting of one of 27 aglycones, known as anthocyanidins. The anthocyanidins usually contain one sugar moiety, which is commonly conjugated to the C3 hydroxyl group in the C-ring, making them glycosides. Sugar moieties such as glucose, galactose, rhamnose, arabinose, and xylose are commonly found in anthocyanins. With the exception of 3-deoxyanthocyanins, anthocyanins exist almost exclusively in a glycosylated form. Their aglycone counterparts are not stable and are rarely found in nature. Six anthocyanidins, namely cyanidin, pelargonidin, delphinidin, petunidin, peonidin, and malvidin, are particularly prevalent in nature and account for approximately 90% of all anthocyanins [18,19]. Isolated anthocyanins are highly unstable and prone to degradation. Their stability can be affected by various factors, such as the pH; storage temperature; chemical structure; concentration; light; oxygen; solvents; and presence of enzymes, flavonoids, proteins, and metallic ions [20].

Anthocyanins are widely distributed in nature and are primarily responsible for the red, blue, and purple colors seen in vegetables, fruits, and their derivatives [21]. Berries, red wine, vegetables, and other fruits are the primary sources of anthocyanins [22], with levels varying considerably among different species. These levels are primarily influenced by plant genotypes and, to a lesser extent, by agricultural practices, growing area, seasonal variability, climatic conditions, temperature and light exposure, ripening stage, and harvesting time, as well as the methods adopted for processing and storage [23]. The daily average intake of anthocyanins is estimated to range from several milligrams to hundreds of milligrams; however, its evaluation is imprecise and depends on factors such as diet, gender, food intolerances in individuals, and the quantities of anthocyanins present in foods [24]. The relative bioavailability of anthocyanins has recently been suggested to be approximately 12% [25].

Anthocyanin pigments have a significant impact on the sensory properties of food, making them desirable for use as food colorants [3]. However, beyond their colorant capacity, anthocyanins may offer numerous health benefits to consumers. There is a growing body of epidemiological and clinical evidence supporting the notion that anthocyanins, as part of a diet rich in fruits and vegetables, play an important role in countering the onset and progression of several disease pathologies, particularly cardiovascular, metabolic, and neurodegenerative diseases, as well as certain types of cancers. The health benefits of anthocyanins are attributed to their free-radical scavenging capacity and their ability to modulate inflammatory cytokine signaling, which is associated with maintaining homeostatic balance in the body [6,26,27].

**Figure 1:**
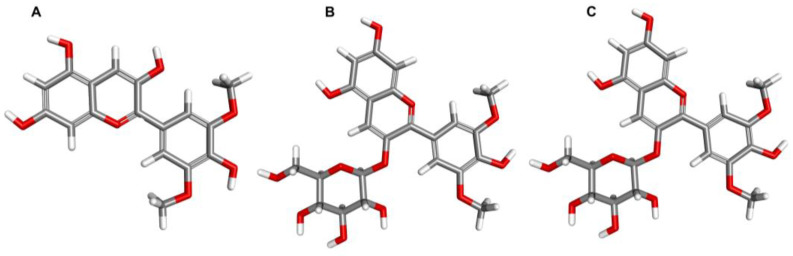
The structure of malvidin (**A**) and its selected glycosides: malvidin-3-glucoside (**B**) and malvidin-3-galactoside (**C**) (https://pubchem.ncbi.nlm.nih.gov/ (accessed on 22 March 2023)).

Malvidin is one of the six most prominent anthocyanins and a member of the *O*-methylated anthocyanidin family. In nature, it is mainly found in its glycosylated form, such as malvidin-3-glucoside and malvidin-3-galactoside, with a sugar moiety attached at position 3 on the C-ring (Figure 1). The solubility of malvidin in water is higher than in methanol and ethanol, likely due to its higher static dipole moment in water [28]. However, this solubility decreases as the degree of acylation increases [29]. Syringic and 4-hydroxybenzoic acids are the primary metabolites of malvidin [30]. When subjected to high pH conditions (pH > 7), acylated malvidin breaks down and releases syringic acid [31].

Malvidin exhibits a visible purple color, is abundantly found in blue-colored flowers [32], and serves as the major red pigment in red wine [33]. The distribution of malvidin glycosides varies across different plant species, with malvidin 3-glucoside and malvidin 3-galactoside being most abundant in fruits and malvidin 3,5-diglucoside in flowers [34]. Grapes predominantly contain malvidin-3-glucoside [25]. The sugar moieties of malvidin glycosides are found at approximately 7% in foods. Malvidin plays a predominant role in regulating both short- and long-term cellular activities and exhibits significant antioxidant properties [6]. Rahman et al. conducted a study on individual anthocyanins and found that delphinidin, isolated from blueberry extracts, has the highest ability to scavenge superoxide species, followed by petunidin, malvidin, cyanidin, peonidin, and pelargonidin, all at concentration of 1 µM. The same trend was observed in their ability to capture peroxynitrite radicals at the same concentration [35]. Table 1 provides a list of the dietary sources of malvidin, which is one of the six major anthocyanins.

## 4. Antioxidant Properties of Malvidin and Their Glycosides

Free radicals are reactive species that can be produced through natural metabolic processes or external sources. They can be derived from oxygen (such as hydroxyl, peroxyl, and superoxide) or nitrogen (such as nitric oxide and peroxynitrite). In addition, there are even-numbered free radical species, such as H_2_O_2_ and lipid peroxide. The accumulation of these radicals can be toxic to cells and trigger reactions such as the oxidation of cellular components, including nucleic acids, proteins, and lipids. This condition is known as oxidative stress, resulting from an imbalance between free radical production and neutralization [51,52]. Anthocyanins have been shown to possess the ability to scavenge free radicals, particularly harmful oxidants such as reactive oxygen and nitrogen species (ROS and RNS) [34,53].

The unique structure of the flavylium cation (AH+) gives anthocyanins distinct antioxidant properties [34,54]. These compounds can neutralize reactive radical species through a single electron transfer (SET) reaction or by a hydrogen atom transfer (HAT) mechanism. In SET, the antioxidant donates an electron to the free radical to neutralize it, while in HAT, the antioxidant donates a hydrogen atom to the free radical that then stabilizes the radical. Both mechanisms usually occur simultaneously, and the reaction mechanism is determined by the structure, solubility, partition coefficient, and solvent polarity of the antioxidant [51,55,56]. Molecules such as anthocyanins have been reported to have the ability to modulate oxidative stress [34,57,58]. In vitro studies demonstrating the antioxidant potential of malvidin and its glycosides are presented in Table 2.

Emandi et al. conducted an in vivo study on rats to investigate the effect of malvidin on renal ischemia–reperfusion injury, which is caused by oxidative stress. The authors found that the administration of malvidin resulted in the increased activity of antioxidant enzymes, such as catalase (CAT) and superoxide dismutase (SOD), as well as decreased levels of the oxidative stress marker malondialdehyde (MDA), compared to the reperfusion injury group. Based on these results, the authors suggest that consumption of malvidin may have a protective effect against acute kidney injury induced by renal reperfusion injury, partly by inhibiting oxidative stress in renal tissues [65].

In summary, malvidin and its glycosides exhibit potent antioxidant activity by donating an electron or hydrogen atom to neutralize free radicals. In addition to this mode of action, they also inhibit enzymes involved in the production of ROS; upregulate or protect antioxidant defenses; and induce antioxidant enzymes such as glutathione peroxidase (GPx), CAT, and SOD, which decompose harmful compounds. Furthermore, studies have shown that malvidin and its glycosides can inhibit the expression of the xanthine oxidase (XO) enzyme. Overall, these findings suggest that malvidin and its glycosides have the potential to block oxidative stress, indicating their usefulness as an antioxidant agents.

## 5. Anti-Inflammatory Properties of Malvidin and Their Glycosides

Highly reactive free radicals can cause damage to cellular and tissue components by oxidizing nucleic acids, proteins, and lipids, resulting in inflammation. Studies have shown that an increase in ROS production leads to the generation of more pro-inflammatory markers. Additionally, experimental evidence has demonstrated that ROS activate the NF-κB pathway, which triggers the transcriptional activation of genes related to inflammatory response [66,67].

In the human body, inflammation is a crucial response, but it is also involved in the pathogenesis of various diseases, such as microbial and viral infections; exposure to allergens; autoimmune disorders; and chronic diseases. Upon activation, innate immune cells secrete proinflammatory cytokines and chemokines, which trigger the production of ROS/RNS. These molecules initiate signaling cascades that further stimulate the release of more proinflammatory agents. However, prolonged inflammation can lead to cell damage or cellular hyperplasia due to excessive ROS production by inflammatory cells. Additionally, during inflammation, ROS can interact with DNA in mitotic cells, leading to permanent genomic mutations, such as point mutations, gene deletions, or gene rearrangements. In response to inflammation, cellular antioxidant systems are activated to counter the overproduction of free radicals by inducing genes involved in DNA repair. However, in cases of chronic inflammation, the rate of ROS-induced DNA damage is significant due to the depletion of cellular antioxidants. This makes cells more prone to transformation and increases the frequency of mutations caused by inflammatory cells [68,69]. Molecules such as anthocyanins have been reported to have the ability to modulate the inflammatory processes [70,71]. In vitro studies demonstrating the ant-inflammatory potential of malvidin and its glycosides are presented in Table 3.

Iban-Arias and colleagues investigated the potential therapeutic effects of malvidin-3-glucoside on inflammation induced by the inflammasome in vitro and in a mouse model of chronic unpredictable stress. Their findings demonstrated that malvidin-3-glucoside targets the inflammasomes NLRP3, NLRC4, and AIM2, leading to a reduction in caspase-1 and IL-1 protein levels in murine primary cortical microglia and the brain. The demonstrated beneficial effect of malvidin includes its ability to counteract anxiety and depression. The study also suggests the potential of malvidin-3-glucoside in mitigating LPS-induced inflammation in vitro, particularly in the context of bacterial-mediated inflammation [80]. Moreover, in a mouse model, malvidin was found to inhibit ROS-dependent NLRP3 inflammasome activation, leading to a decrease in serum pro-inflammatory cytokine secretion and mitochondrial-pathway-mediated apoptosis. Malvidin also increased Bcl-2 protein levels, while decreasing Bax, cytochrome C, and caspase-3 levels. The study demonstrated that malvidin targeted the AMPK-α/UCP2 axis, which restored mitochondrial function and reduced ROS accumulation, ultimately leading to the inhibition of NLRP3 inflammasome activation and mitochondrial apoptosis in a ROS-dependent manner [81].

Dai et al. conducted an in vivo study to examine the effects of malvidin on osteoarthritis in rats induced by monosodium iodoacetate administration. The results showed that malvidin treatment significantly relieved pain in the osteoarthritis rats and reduced the expression of apoptotic markers in chondrocytes. In addition, the upregulation of proinflammatory cytokines IL-1β, IL-6, TNF-α, and matrix metalloproteinases (MMPs) induced by monosodium iodoacetate in cartilage tissues were significantly reversed by malvidin treatment. Malvidin also inhibited the NF-κB pathway through a mechanism independent of NF-κB inhibitor (IκBα) by suppressing p65 nuclear transport in vitro. Overall, the findings suggest that malvidin can effectively attenuate pain and inflammation induced by osteoarthritis by inhibiting the NF-κB signaling pathway, suppressing proinflammatory cytokine expression, and reducing chondrocyte apoptosis [82].

To summarize, malvidin and its glycosides have been shown to possess anti-inflammatory effects through their radical scavenging activities. Various experiments have provided evidence that malvidin and its glycosides can modulate different inflammatory mediators, including protein kinases; transcription factors such as AP-1 and NF-κB; enzymes such as iNOS and COX-2; and cytokines such as IL-1β, IL-6, and TNF-α, which are known to play a central role in the inflammation process. These findings suggest that malvidin and its glycosides could serve as potential therapeutic agents for inflammatory diseases by blocking inflammation.

## 6. The Roles of Malvidin and Its Glycosides in Oxidative Stress and Inflammation-Mediated Chronic Disorders

Oxidative stress and inflammation are two significant physiological processes with close connections that modulate cellular physiological and pathological responses. The body possesses an endogenous defense system to maintain cellular homeostasis by combating both processes. However, prolonged oxidative stress and inflammation can be detrimental to the body’s defense system [83,84]. ROS can activate pro-inflammatory cytokines and the NLRP3 inflammasome, thereby regulating inflammation [85,86]. The findings regarding the antioxidant and anti-inflammatory properties of malvidin and its derivatives provide an insight into their potential molecular mechanisms of action in mitigating oxidative stress and inflammation. Research on malvidin and its glycosides has demonstrated their ability to act as antioxidants through various mechanisms, including scavenging free radicals and anions.

Additionally, studies have found that these molecules can activate the nuclear factor erythroid 2-related factor (Nrf2) pathway, which is sensitive to oxidative stress. Nrf2 is a transcription factor that is normally bound to the actin protein Keap1, but exposure to ROS leads to Nrf2 degradation via the ubiquitin proteasome pathway. However, malvidin and its glycosides stabilize Nrf2, allowing it to accumulate in the nucleus and activate the antioxidant response element (ARE)-regulated target genes. Nrf2 plays a crucial role in regulating endogenous antioxidant defense by upregulating enzymes such as glutathione S-transferase (GST) and peroxidase (GPx), hemeoxygenase-1 (HO-1), CAT, and SOD. The Keap1-Nrf2 system helps protect cellular components from oxidative damage caused by ROS by increasing antioxidant enzyme expression and decreasing sensitivity to oxidative-stress-related inflammatory reactions [87,88]. Additionally, Nrf2 promotes the activation of the pentose phosphate pathway (PPP), leading to NADPH production, which is involved in regenerating reduced glutathione (GSH) from GSH disulfide and maintaining cellular antioxidant levels [89]. Therefore, enhancing Nrf2 induction efficiency provides homeostatic mechanisms for the antioxidant activity of malvidin and its glycosides.

Research has also revealed that these molecules target arachidonic acid. Lipid mediators derived from arachidonic acid, such as prostaglandins produced via COX-2 and leukotrienes via lipoxygenases (LOX), are important targets of anthocyanins. COX enzymes are essential for the conversion of arachidonic acid to prostanoids, which play a significant role in inflammation. LOX uses arachidonic acid as a substrate and catalyzes four different reactions, namely 5*S*, 12*R*, 12*S*, or 15*S* oxygenation. The oxygenated substrates of these enzymes initiate biological reactions, activate cellular signaling through surface receptors, or are metabolized into potent lipid mediators. Previous studies have highlighted the influence of LOX in the inflammatory process [90,91,92].

According to several studies, malvidin and its glycosides have demonstrated inhibitory effects on the NF-κB pathway which plays a crucial role in regulating the inflammatory response. The canonical NF-κB signaling pathway is activated by various inflammatory stimuli, such as exposure to LPS, TNF-α, or IL-1. Activation of the canonical NF-κB pathway involves phosphorylation-dependent activation of the IKKs complex, leading to the degradation of inhibitory IκB proteins by the ubiquitin–proteasome system. This release allows the κB transcription factor to translocate to the nucleus and activate target genes. NF-κB is responsible for the transcription of a wide range of genes involved in the inflammatory response, including chemokines, cytokines, and adhesion molecules, as well as genes that negatively regulate its activity [93,94].

Several studies have suggested that malvidin and its glycosides possess inhibitory effects on the MAPK pathway. Under normal physiological conditions, MAPK signaling pathways play a crucial role in various processes, including cell proliferation, survival, and differentiation. These pathways are activated by extracellular stimuli, such as cytokines, Toll-like receptors (TLRs), and oxidative stress, which trigger the corresponding receptors to transduce intracellular signaling into the nucleus through three primary MAPK cascades. In humans, the extracellular-signal-regulated kinase (ERK) 1/2, *C*-jun *N*-terminal kinase (JNK), and p38 MAPKs are the three main kinases. It is hypothesized that intracellular kinases such as MAPKK, MEK, or MKK and MAPK initiate downstream activation of MAPKs (ERK1/2, JNK, and p38 MAPK) by phosphorylating specific residues in the appropriate protein. Once activated, MAPKs move into nuclei and phosphorylate the relevant transcription factors, thereby regulating gene expression. Activation of the MAPK pathway also leads to the activation of the transcription factor AP-1, which, similar to NF-κB, contains transcriptional regulator binding sites for most inflammatory mediators. AP-1 can also bind promoters of inflammatory mediators independently of NF-κB during inflammation [95,96].

**Figure 2:**
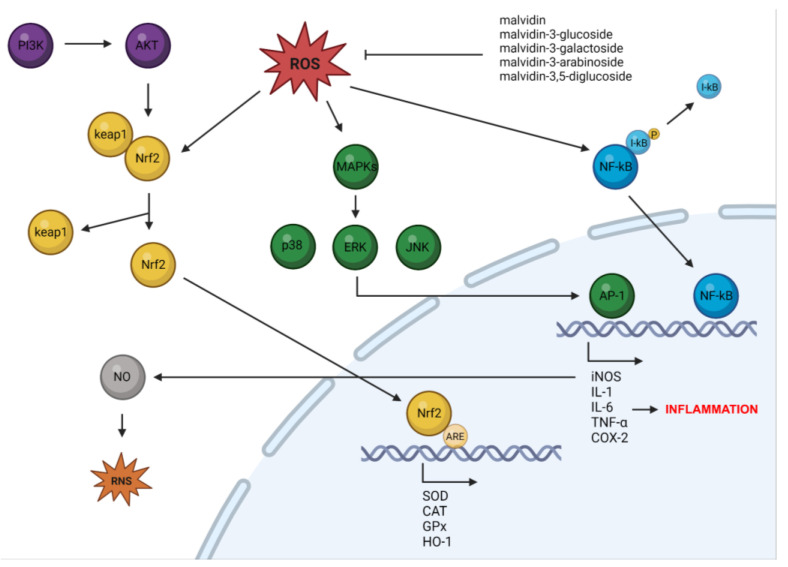
The mechanisms through which malvidin and its glycosides act as antioxidant and anti-inflammatory agents (created by BioRender: https://www.biorender.com/ (accessed on 6 April 2023)).

The antioxidant and anti-inflammatory mechanisms of action of malvidin and its glycosides are presented in Figure 2. Oxidative stress and inflammation are both key causative factors for the onset of several diseases, including cancer, metabolic and cardiovascular disorders, and neurodegenerative diseases.

### 6.1. Anti-Cancerogenic Properties of Malvidin and Their Glycosides

Cancer is a significant cause of death worldwide, with approximately 10 million deaths in 2020, accounting for approximately one in six deaths [97]. Breast, lung, colon, rectum, and prostate cancer are among the most prevalent types. Cancer is a complex process involving at least three stages: initiation, promotion, and progression [98]. Cell-cycle dysregulation is a crucial factor in cancer cell growth. The cell cycle comprises a series of tightly regulated events that enable cell growth and division. Cyclin-dependent kinases (CDKs) are essential components of the cell-cycle machinery that, when activated, facilitate progression from one phase to the next. The regulation of CDKs involves positive regulation by cyclins and negative regulation by CDK inhibitors (CDKIs). In cancer, the cell cycle is often dysregulated through the overexpression of cyclins or the absence of CDKIs [99]. Oxidative stress and inflammation are closely linked to cancer progression. ROS may initiate carcinogenesis directly or through the activation of signaling pathways. Tumor promoters have the ability to attract inflammatory cells and induce them to produce ROS, which is an important characteristic [100,101].

Cancer cells have a modified metabolism to meet the increased energy requirements due to their rapid growth and proliferation. Consequently, they produce more ROS than normal cells to maintain typical subcellular activities, including signal transduction and gene expression [102,103]. Research has established that ROS contribute to tumorigenesis through the activation of oncogenic cell signaling pathways [104]. The phosphoinositide-3-kinase (PI3K)/AKT and MAPK pathways, which promote cell survival, proliferation, metabolism, nutrient uptake, and inflammation, are commonly associated with cancer [105,106]. H_2_O_2_ inhibits protein tyrosine phosphatase 1B (PTB1B), which, in turn, prevents the dephosphorylation of epidermal growth factor receptor (EGFR), leading to the activation of downstream PI3K/AKT and RAS-MEK-ERK (ERK/MAPK) pathways [104]. The GTPase RAS is frequently implicated in tumorigenesis by activating MAPK pathways and regulating transcription. Specifically, K-RAS activates JNK, ERK, and p38 signaling pathways, with the latter also contributing to ROS production via NADPH oxidase 1 (NOX1) [107]. It is hypothesized that ROS may have varying effects on tumorigenesis, either enhancing, inhibiting, or regulating it, depending on the activation of MAPK pathways in different cancers [108]. However, the excessive accumulation of ROS is typically detrimental to cancer cells [109]. Despite contributing to cancer progression, high levels of ROS can also induce cell death in cancer cell lines [85,86]. Inflammation is regulated by ROS through the activation of pro-inflammatory cytokines and the NLRP3 inflammasome [56].

Inflammation plays a significant role in the development and progression of cancer by promoting cell proliferation, survival signaling, angiogenesis, invasion, and metastasis. Pro-inflammatory cytokines such as IL-1, IL-6, and TNF-α are elevated in both the plasma and cells of cancer patients [110]. These cytokines activate NF-κB and signal transducers and activators of transcription 3 (STAT3), which are involved in cancer growth [111,112,113]. TNF-α induces tumorigenesis through ROS production, which can cause DNA damage [114]. Mutant p53 has been shown to alter TNF-α signaling to favor NF-κB activation [115]. NF-κB can increase oncogenic K-RAS levels in a positive feedback loop, further correlating chronic inflammation with cancer progression [116].

**Figure 3:**
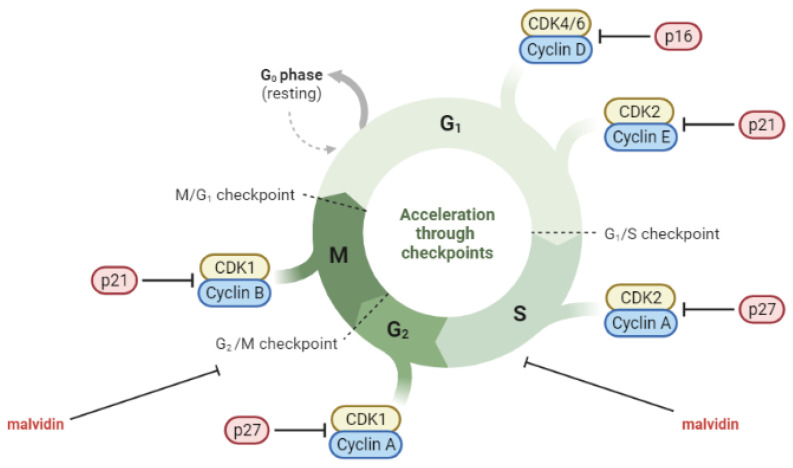
Cell-cycle deregulation in cancer cells and the role of malvidin (created by BioRender: https://www.biorender.com/ (accessed on 10 April 2023)).

Molecules such as malvidin and its glycosides have been reported to be able to modulate the carcinogenesis processes [117,118]. Two separate in vitro studies conducted by Lin et al. and Wang et al. investigated the anticancer effects of malvidin-3-galactoside on hepatocellular carcinoma. The studies found that the compound inhibited cell proliferation and colony formation, induced cell-cycle arrest and apoptosis, and suppressed migration and invasion potential by regulating the expression of MMPs. The anticancer effects of malvidin-3-galactoside were associated with the inhibition of the PI3K/AKT, MAPK, and MMP pathways. These findings suggest that malvidin-3-galactoside may have potential for preventing liver cancer by modulating proliferation, apoptosis, migration, and invasion-related pathways [119,120]. In a study conducted by Xu et al., the anticancer activity of malvidin was evaluated using the human colorectal cancer cell line. Their findings showed that malvidin exhibited cytotoxic effects against the cells by inhibiting colony formation and inducing apoptosis. Malvidin was also found to induce G2/M cell-cycle arrest and inhibit cell-cycle-related proteins [121]. Baba et al. investigated the potential of malvidin to target the transcription factor STAT-3 in the oral cancer cell line. Their results indicated that malvidin acts as a STAT-3 inhibitor, suppressing its phosphorylation and nuclear translocation, which, in turn, induced cell-cycle arrest and apoptosis through mitochondrial-mediated pathways [122]. Dahlawi conducted a study on the effects of malvidin on two human leukemia cell lines. The results revealed that malvidin inhibited cell proliferation and induced apoptosis in both leukemia cell lines, as evidenced by caspase-3 activation. Malvidin also caused cell-cycle arrest at the S phase in both cell lines [123]. Similarly, Hyun and Chung reported on the cytotoxic effect of malvidin on the human monocytic leukemia cell line. Malvidin induced apoptosis and arrested the G2/M phase of the cell cycle [124]. Ouanouki et al. conducted a study to investigate the impact of malvidin on TGF-β-induced epithelial–mesenchymal transition (EMT) and its underlying mechanism. EMT is a process that allows benign tumor cells to infiltrate surrounding tissues. The researchers treated human glioblastoma cell line with malvidin before or together with or after the addition of TGF-β. They found that malvidin inhibited TGF-β-induced EMT by affecting both the TGF-β Smad and non-Smad signaling pathways. This inhibitory effect altered the expression of the EMT mesenchymal markers fibronectin and Snail and significantly reduced the migration of cells [125]. The role of malvidin and its glycosides in the regulation of cell cycles in cancer cells is presented in Figure 3.

The anti-carcinogenic effects observed in vitro have been validated in vivo. In a study by Sakthivel et al., mice with Dalton’s lymphoma were treated with malvidin for 10 consecutive days, starting from the day of tumor induction. Treatment with malvidin led to a significant reduction in tumor volume and an increase in white blood cell count. The treatment also maintained body weight and the hemoglobin level, and decreased levels of liver enzymes such as aspartate aminotransferase (AST), alanine aminotransferase (ALT), alkaline phosphatase (ALP), and gamma-glutamyl transferase (GGT) were observed. Additionally, malvidin reduced the levels of inflammatory mediators and cytokines such as TNF-α and IL-6, which are molecular targets for cancer prevention. A decrease in the level of ROS, such as NO, was also observed. A histopathological examination showed altered morphological changes in tumor tissue and the alleviation of hepatic architecture due to Dalton’s lymphoma. An immunohistochemical analysis revealed the inhibition of iNOS [126]. Lin et al. reported that malvidin-3-galactoside induced apoptosis in liver tumor cells in mice [119]. In another study, Cheng et al. investigated the effects of malvidin-3-galactoside on gut microbiota in liver-cancer mice. The mice were fed diets supplemented with malvidin-3-galactoside for three weeks. The results showed an increase in the abundance of *Verrucomicrobiaceae* and *Ruminococcus*, as well as anti-inflammatory bacteria such as *Akkermansia* and *Sutterella*. On the other hand, a decrease in the abundance of proinflammatory bacteria such as *Dorea*, *Coprobacillus*, *Clostridium*, *Streptococcus*, and *Oscillospira* was observed. Malvidin-3-galactoside was found to enhance signal transduction, membrane transport, and cell motility, as well as induce cell death. The study suggests that malvidin-3-galactoside may have significant impacts on the structure and metabolic function of gut microbiota in liver-cancer mice [127].

In conclusion, abovementioned studies provide evidence of the significant anticancer effects of malvidin and its glycosides, and such effects may be attributed to their antioxidative and anti-inflammatory mechanisms of action. However, there is currently a lack of unequivocal human trials allowing for the assessment of the anticancer effect of malvidin and its glycosides.

### 6.2. Antidiabetic Properties of Malvidin and Its Glycosides

According to a 2019 report, diabetes and diabetes-related kidney disease were responsible for an estimated two million deaths. Diabetes is a disorder characterized by elevated glucose levels, and it has multiple subtypes [128,129]. Prolonged high levels of glucose and free fatty acids can lead to severe complications, including organ failure and tissue damage [130]. It has been linked to damage in the nervous system, vascular endothelium, and kidneys that can be attributed to stress-activated signaling pathways such as NF-κB and MAPKs and other stress-activated protein kinases.

The production of ROS is triggered by hyperglycemia and is also believed to play a role in the pathogenesis of disease [131]. Elevated glucose levels are known to induce oxidative stress by upregulating mitochondrial ROS, causing protein glycation, and triggering glucose autooxidation. These processes can potentially impair enzyme activity and cellular function. In addition, high levels of free fatty acids can lead to mitochondrial uncoupling and β-oxidation, also resulting in oxidative stress. Moreover, advanced diabetes is associated with reduced levels of important antioxidants such as vitamin E and α-lipoic acid, as well as SOD, an enzyme that plays a crucial role in inactivating the superoxide radical [132]. Superoxide radicals, in turn, activate multiple pathways, including enhanced polyol formation, increased hexosamine pathway flux, and the activation of the PKC isoform [133].

There is a growing body of evidence indicating a correlation between moderate inflammation and the onset of type 2 diabetes mellitus (T2DM). Furthermore, there is a possibility that inflammation may play a role in the development of diabetes. Research has demonstrated that inflammatory cytokines can interfere with insulin-signal transduction, leading to insulin resistance [134]. Additionally, genes such as IL-1α, IL-1β, IL-6, and TNF-α are continually activated in individuals with diabetes. Inflammation primarily targets immune cells and endothelial cells in diabetic patients [135]. In addition, inflammation could potentially trigger the degradation of β-cells. Increased production of IL-1β, IL-6, and IL-8 in pancreatic islets due to glucotoxicity and lipotoxicity in diabetes can lead to a reduction in insulin gene transcription and an increase in macrophages in the pancreas, ultimately leading to apoptosis of β-cells [136].

Molecules such as malvidin and its glycosides have been reported to be able have beneficial effects on diabetes [137,138]. In a study by Herrera-Balandrano et al., it was demonstrated that exposing human hepatocarcinoma cells to high levels of glucose led to a significant increase in hepatic oxidative stress, of up to six times, and a decrease in cell viability. However, pretreatment with malvidin, malvidin-3-glucoside, and malvidin-3-galactoside resulted in a significant reduction of ROS and increase in cell viability. These pretreatments were also able to reduce the expression levels of enzymes involved in gluconeogenesis and lipogenesis and enhance those involved in glycogenolysis and lipolysis via the AMPK signaling pathway in cells, effectively inhibiting hyperglycemia and hyperlipidemia [139]. Huang et al. reported that malvidin, malvidin-3-glucoside, and malvidin-3-galactoside could protect human retinal capillary endothelial cells from high-glucose-induced injury by decreasing ROS and increasing CAT and SOD enzyme activity. The study also revealed that malvidin significantly inhibited NOX4 expression induced by high glucose, while malvidin-3-galactoside downregulated NOX4. Furthermore, malvidin, malvidin-3-glucoside, and malvidin-3-galactoside affected NO levels and inhibited high-glucose-induced intercellular adhesion molecule-1 (ICAM-1) and NF-κB expression. Malvidin-3-galactoside also influenced angiogenesis by decreasing the level of vascular endothelial cell growth factor (VEGF) and inhibiting the PI3K/AKT pathway [140]. In addition, Huang et al. found that pretreatment of human umbilical vein endothelial cells with malvidin, malvidin-3-glucoside, and malvidin-3-galactoside significantly improved high-glucose-induced damage by enhancing endogenous antioxidant SOD and HO-1, reducing ROS generation and NOX4 expression, and increasing cell viability. They also induced vasodilation by increasing the vasodilator NO and its promoters’ endothelial NO synthase (eNOS) and peroxisome proliferator-activated receptor (PPAR) levels, while decreasing the vasoconstrictor angiotensin-converting enzyme (ACE), XO-1, and low-density lipoprotein (LDL) levels. These bioactivities were linked to the activation of the PI3K/AKT signaling pathway and the breakdown of the PKC pathway [141].

Clinical trials (https://clinicaltrials.gov (accessed on 14 March 2023)) analyzing the effects of anthocyanins, including malvidin and its glycosides, on patients with T2DM have been reported on the clinicaltrials.gov website. In the first study, patients diagnosed with T2DM were administered Medox, two capsules with anthocyanins, twice daily for 12 weeks. Each capsule contained 80 mg of anthocyanins, including 3.0% of malvidin-3-glucoside, malvidin-3-galactoside, and malvidin-3-arabinoside. The trial showed a significant decrease in serum LDL cholesterol, triglycerides, apolipoprotein B-48, and apo C-III and an increase in high-density lipoprotein (HDL) cholesterol. Moreover, patients in the anthocyanin group had higher total radical-trapping antioxidant parameter and ferric-ion-reducing antioxidant power values than those in the placebo group. Concentrations of 8-iso-prostaglandin F2α, 13-hydroxyoctadecadienoic acid, and carbonylated proteins were significantly lower in patients in the anthocyanin group than in the placebo group. Supplementation with anthocyanin also reduced fasting plasma glucose and homeostasis model assessment for insulin resistance index and increased serum adiponectin and β-hydroxybutyrate concentrations compared with placebo supplementation. The trial was registered as NCT02317211 [142]. In another study, Medox capsules were administered to 160 participants with prediabetes or newly diagnosed T2DM, with a daily supplementation of 320 mg anthocyanins for 12 weeks. The study showed significant increases in serum adipsin and decreases in visfatin between the anthocyanins and placebo groups. Furthermore, improvements in HbA1c, apolipoprotein A-1, and apolipoprotein B were observed in response to the anthocyanin intervention. This study was registered as NCT02689765 [143]. The following study investigated the potential effects of a standardized extract called Mirtoselect^®^ on glucose metabolism in patients with T2DM. Mirtoselect^®^ is derived from the berries of *Vaccinium myrtillus* and contains 36% (*w*/*w*) of anthocyanins, including malvidin-3-galactoside, malvidin-3-glucoside, and malvidin-3-arabinoside. Participants with T2DM who were managing their condition through diet and lifestyle alone were given a single oral capsule of either 0.7 g of standardized bilberry extract or a placebo, followed by a polysaccharide drink (equivalent to 75 g of glucose) after a 2-week washout period. The results showed that ingestion of the bilberry extract led to a significant decrease in both glucose and insulin levels compared to the placebo. This trial was registered as NCT01245270 [144]. These findings demonstrate the potential beneficial effects of anthocyanin supplementation in individuals with T2DM.

In conclusion, the abovementioned studies provide evidence of the significant antidiabetic effects of malvidin and its glycosides, which may be attributed to their antioxidative and anti-inflammatory mechanisms of action. The inclusion of diet-induced anthocyanins, such as malvidin and its glycosides, may present a natural alternative for improving glycemic control in individuals with T2DM. However, further research is necessary to fully comprehend the specific ways in which anthocyanins, including malvidin and its glycosides, can contribute to the management of T2DM.

### 6.3. Cardioprotective Effect of Malvidin and Their Glycosides

Cardiovascular diseases (CVDs) are a major cause of mortality worldwide, resulting in an estimated 17.9 million deaths annually. CVDs refer to a group of disorders that affect the heart and blood vessels, including conditions such as myocardial infarction, heart failure, stroke, peripheral arterial disease, arrhythmia, and atrial fibrillation. In addition, CVDs have been linked to the development of dementia and loss of daily living function. Overall, CVDs are a significant cause of mortality, morbidity, disability, and loss of function [145,146].

Oxidative stress is a significant contributor to the development of atherosclerosis, which is the primary cause of cardiovascular diseases. Atherosclerosis involves the formation of fibrofatty lesions in the artery wall. The development of atherosclerotic lesions is likely initiated by the oxidation of LDL, which is responsible for carrying cholesterol through the blood. Oxidized LDL activates the endothelium and triggers an immune system response, leading to the migration of inflammatory cells such as monocytes and T cells into the arterial intima. Phagocytosis of the oxidized LDL by macrophages causes the release of ROS and pro-inflammatory markers, which lead to further LDL oxidation [147]. This cycle perpetuates the progression of atherosclerosis [148].

The dysfunction of the endothelium may be caused by the presence of ROS generated by the electron transport chain of the mitochondria. This includes mitochondrial ROS and ROS generated by NOX, which have been shown to positively regulate each other. CVDs have been linked to ROS produced by NOX enzymes expressed in vascular tissue. Specifically, NOX4 is involved in regulating vascular smooth-muscle cells, fibroblasts, and the differentiation and migration of cardiac cells. Overexpression of NOX4 can lead to negative effects on cells due to the increased production of H_2_O_2_. Moreover, in a healthy state, NO plays a crucial role in regulating endothelial function. However, under conditions of elevated ROS levels, such as when superoxide ions are present, NO can become oxidized and transform into peroxynitrite, a potent oxidizer that causes further oxidation and cell damage [148,149,150].

Malfunctions in several enzymes related to ROS, including XO, LOX, SOD, and GPx, may also predispose to CVDs. XO participates in the regulation and dysregulation of the endothelium. The generation of ROS by XO can interact with superoxide ions, resulting in the formation of peroxynitrite and inducing cellular harm. Furthermore, the ROS produced by XO can directly interact with the epidermal growth factor receptor (EGFR), contributing to vascular remodeling and CVDs. LOX also contributes to the development of CVDs [148]. Specifically, 5-LOX is of interest due to its involvement in the activation of inflammatory cells. In response to oxidative stress, 5-LOX can be upregulated; meanwhile, the downregulation of 5-LOX has shown positive effects on myocardial infarction [148,151]. SOD also plays a role in vascular disease under oxidative stress. SOD is responsible for converting superoxide ions into H_2_O_2_, with SOD1 being crucial in maintaining endothelial function by preserving NO availability [152,153]. GPx activity is frequently used as a marker for disease and ROS levels in individuals with coronary artery disease and atherosclerosis [154]. GPx safeguards erythrocytes from oxidation and subsequent damage [155]. Research has shown an inverse relationship between erythrocyte GPx activity and coronary artery disease, similar to that observed in atherosclerosis [154].

Cytokines are known to play a crucial role in the development and progression of atherosclerosis and CVDs. For instance, interferon (IFN)-γ stimulates the expression of the pro-inflammatory phenotype (M1) of macrophages, leading to the formation of arterial plaques and cell apoptosis, which results in lipid expulsion into adjacent plaque regions. Interleukin IL-1β, in addition to IFN-γ, can also activate the M1 macrophage phenotypes in an auto-inflammatory way or stimulate other pro-inflammatory genes. Additionally, oxidized LDL can increase the expression of IL-1β, resulting in increased inflammation within the atherosclerotic plaque region. The chemokine (C-C motif) ligand 2 (CCL-2) plays a role in the development of atherosclerotic plaques through the activation of PKC, ERK1, and NF-κB signaling pathways. Studies have shown that cells lacking CCL-2 produce smaller plaques [156].

Molecules such as malvidin and its glycosides have been reported to have beneficial effects on CVDs [157,158]. Del Bo et al. conducted a study to investigate the potential of malvidin-3-glucoside to reduce inflammation-driven adhesion of monocytes to endothelial cells and the secretion of cell-adhesion molecules, including E-selectin and vascular cell adhesion molecule 1 (VCAM-1). The adhesion was induced by TNF-α. The results showed that malvidin-3-glucoside reduced monocytes’ adhesion to endothelial cells and significantly reduced E-selectin levels, but not VCAM-1 levels. The study concluded that malvidin-3-glucoside had the potential to resolve inflammation-driven adhesion by reducing E-selectin concentrations and that it was effective at physiologically relevant concentrations [159]. Quintieri et al. investigated the effects of malvidin on cardiovascular function, using isolated and Langendorff-perfused rat hearts. The authors observed that malvidin treatment resulted in negative inotropic and lusitropic effects, as well as coronary dilation. The mechanism-of-action analysis revealed that the cardiac effects of malvidin required the activation of the PI3K/NO/cGMP/cGMP-dependent protein kinase pathway and were associated with increased intracellular guanosine-3′,5′-cyclic monophosphate (cGMP) and the phosphorylation of eNOS, PI3K-AKT, ERK1/2, and glycogen synthase kinase 3 beta (GSK-3β). AKT and eNOS phosphorylation were confirmed in endothelial cells. Malvidin acted as a postconditioning agent, being able to elicit cardioprotection against ischemia/reperfusion damages [160].

Wei et al. investigated the effect of malvidin on myocardial infarction induced by isoproterenol in rats. The study demonstrated that malvidin has significant cardioprotective effects by restoring the defensive activities of endogenous antioxidants, such as CAT, SOD, and GSH, and by reducing the levels of lipid peroxidation and serum marker enzymes lactate dehydrogenase (LD) and creatine kinase (CK). Malvidin also improved the histopathological changes and impaired mitochondria in a cardiac necrosis model stimulated by isoproterenol. Additionally, the results showed that the nuclear translocation of Nrf-2 and subsequent HO-1 expression might be associated with the activation of the NF-kB pathway [161].

An ongoing human clinical trial (https://clinicaltrials.gov (accessed on 17 March 2023)) is investigating whether exposure to anthocyanins, including malvidin, can lower the levels of various markers associated with CVDs risk. A study was conducted to investigate the effects of daily consumption of freeze-dried blueberry powder containing *Vaccinium* species—which are a source of anthocyanins, including malvidin [162,163]—on blood pressure and arterial stiffness in postmenopausal women with pre- and stage-one-hypertension. After eight weeks, the blueberry-powder group showed significantly lower systolic and diastolic blood pressure and brachial–ankle pulse wave velocity compared to baseline levels, as well as higher levels of NO. This suggests that daily blueberry consumption may reduce cardiovascular disease risk by reducing blood pressure and arterial stiffness, possibly due to the increased NO production. The trial was registered as NCT03370991 [164]. This finding demonstrates the potential beneficial effects of anthocyanin supplementation in individuals with hypertension.

In conclusion, the abovementioned studies provide evidence of the significant cardioprotective effects of malvidin and its glycosides, and such effects can be attributed to their antioxidative and anti-inflammatory mechanisms of action. The inclusion of diet-induced anthocyanins, such as malvidin and its glycosides, may present a natural alternative for prevention against the development of CVDs. However, further research is necessary to fully comprehend the specific ways in which anthocyanins, including malvidin and its glycosides, can contribute to the protection of CVDs.

### 6.4. Neuroprotective Effects of Malvidin and Its Derivatives

Dementia, affecting over 55 million individuals globally, arises from a range of brain diseases and injuries. The primary cause is Alzheimer’s disease (AD), accounting for 60–70% of cases [165]. Advanced age is a significant risk factor for cognitive impairment and neurodegenerative conditions such as Alzheimer’s and Parkinson’s diseases [166].

Aging leads to increased oxidative stress in the nervous system, impairing nerve regeneration and function [167]. Specifically, voltage-gated potassium (K+) channel sub-family B member 1 (KCNB1) is shown to be subjected to moderate oxidation in aged people, causing hippocampal functional impairment. In AD or following a trauma, the oxidation of KCNB1 is aggravated, resulting in marked neurodegeneration [148]. Moreover, inflammation is detected in the human central nervous system. Increased levels of inflammatory cytokines, including TNF-α, IFN-γ-induced protein 10 (IP-10), and IL-8, have been observed in the cerebrospinal fluid of aging individuals [168].

Molecules such as malvidin and its glycosides have been reported to be able to impact beneficial effects on neurodegenerative disease [169,170]. In a study by Lin and colleagues, the potential neuroprotective effects of malvidin against hypoxia in glial cells were explored. The cells were initially treated with malvidin under normoxic conditions and subsequently exposed to hypoxia, using sodium dithionite in an anaerobic incubator. The results indicated that preincubation with malvidin led to increased CAT activity and GSH concentration following hypoxia treatment, compared to control samples without anthocyanidin preincubation. Additionally, cells preincubated with malvidin showed higher SOD activities [171]. In another study by Zhao et al., the effects of malvidin on a murine microglial cell line were investigated, showing that it prevented mitochondrial dysfunction and accumulation of ROS, decreased lipid peroxidation, and increased antioxidant enzyme activity in the cerebrum [81]. In the same study, malvidin was also administered to mice with sepsis-associated encephalopathy, resulting in the restoration of neurobehavioral retardation; decreased levels of serum S100 calcium-binding protein β (S100β) and neuron-specific enolase (NSE); sustained cerebrum morphological structure, improved blood–brain barrier integrity, with elevated tight junction proteins; and decreased Evans blue leakage. Ultimately, malvidin protected mice from brain injury [81].

Giliani et al. conducted a study to investigate the antioxidant properties of malvidin against aluminum chloride (AlCl3)-induced neurotoxicity in rats. After 61 days of treatment, the rats’ brains were examined using a neurochemical assay. The results showed that malvidin improved the behavioral parameters affected by AlCl3. A biochemical analysis demonstrated that the oral administration of malvidin had neuroprotective effects through the regulation of antioxidant levels and neuroinflammation in the rats exposed to AlCl_3_. These findings suggest that malvidin possesses antioxidant activity by inhibiting acetylcholinesterase and regulating oxidative stress in neuronal cells [172]. Lapi et al. conducted a study to investigate the protective effects of malvidin against damage caused by bilateral common carotid artery occlusion and reperfusion in rat pial microcirculation. In hypoperfused rats, the occlusion and reperfusion caused a decrease in arteriolar diameter, an increase in microvascular leakage and leukocyte adhesion, and decreased capillary perfusion and red blood cell velocity, as well as marked neuronal damage and ROS generation. However, malvidin administration induced arteriolar dilation in a dose-related manner, reduced microvascular leakage and leukocyte adhesion, protected capillary perfusion and red blood cell velocity, prevented neuronal damage, and decreased ROS generation. The study suggested that malvidin’s effects were mediated by eNOS activation and scavenger activity, and NO synthase inhibition significantly attenuated malvidin’s effects on arteriolar diameter. Furthermore, an increase in eNOS and *p*-eNOS expression and a decrease in MMP-9 activity were observed after malvidin’s administration [173].

Clinical trials on humans (https://clinicaltrials.gov (accessed on 21 March 2023)) have investigated the effects of anthocyanins, including malvidin, on memory disorders and dementia patients. A clinical trial registered as NCT03419039 investigated the effects of anthocyanins, including malvidin, on cognitive functioning in individuals at risk for dementia. The trial included 206 participants with mild cognitive impairment or cardiometabolic disorders who received 320 mg/day of naturally purified anthocyanins, including malvidin, for 24 weeks. Although there was no significant difference in episodic memory at the end of the study, statistically significant differences in slopes were observed [174]. Another trial, registered as NCT01746303, evaluated the effects of long-term supplementation of blueberry powder containing *Vaccinium* species, a source of anthocyanins, including malvidin [162,163], on older adults with cognitive complaints. The trial included 94 participants who received 25 g of blueberry powder daily for 24 weeks. The tested group reported fewer cognitive symptoms and showed improved memory discrimination, indicating improved cognition. The cognitive benefit was associated with the presence of urinary anthocyanins [175]. These findings demonstrate the potential beneficial effects of anthocyanin supplementation in individuals with dementia.

In conclusion, the abovementioned studies provide evidence of the significant neuroprotective effects of malvidin and its glycosides that may be attributed to their antioxidative and anti-inflammatory mechanisms of action. The inclusion of diet-induced anthocyanins, such as malvidin and its glycosides, may present a natural alternative for prevention against the development of neurological disorders. However, further research is necessary to fully comprehend the specific ways in which anthocyanins, including malvidin and its glycosides, can contribute to the protection of brain injury.

## 7. Potential Applications of Malvidin and Its Glycosides

The color of a food product plays a significant role in its overall appeal. Anthocyanins, including malvidin and its glycosides, offer appealing colors of red, purple, and blue. They are naturally abundant and pose no harm to consumers, making them desirable as natural color additives. However, their limited stability, especially in comparison to artificial dyes, has restricted their widespread utilization [6,56]. The chemical stabilization of anthocyanins is currently the primary focus of recent studies due to their abundant potential applications, beneficial effects, and use as an alternative to artificial colorants [20,176]. Pazmiño-Durán et al. conducted a study to assess the anthocyanin pigment content and profile found in *Oxalis triangularis* leaves, with the aim of exploring its potential as a natural food coloring agent. The results revealed a monomeric anthocyanin content of 195 mg per 100 g of leaves, based on malvidin-3,5-diglucoside. These findings indicate that *Oxalis triangularis* possesses an appealing hue and high anthocyanin content and is safe for consumption, making it a promising candidate as a natural colorant source [177]. In addition, Mojica et al. proposed that the utilization of anthocyanins extracted from black bean coats, particularly malvidin-3-glucoside, hold promise as a natural alternative for food coloring purposes. The study revealed the presence of 32 mg of anthocyanins per gram of dry extract. These findings indicate that black-bean coats also have the potential to serve as a viable source of natural food colorants [178].

**Figure 4:**
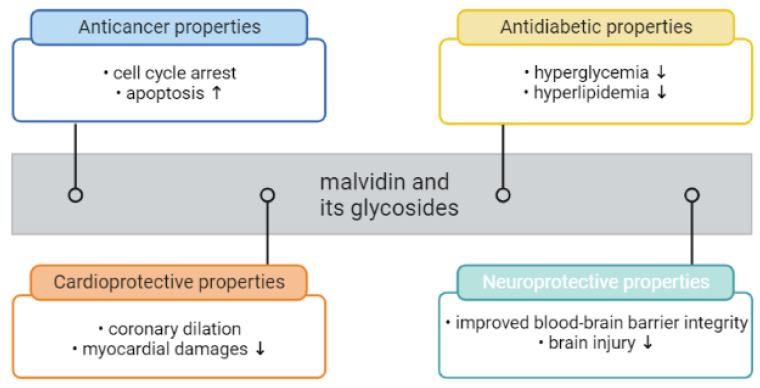
Health-benefit potential of malvidin and its glycosides (created by BioRender: https://www.biorender.com/ (accessed on 8 May 2023)). Downward arrow (↓) indicating decrease, upward arrow (↑) indicating increase.

Besides the color attributes, anthocyanins, including malvidin and its glycosides, have been reported to be beneficial to health (Figure 4), possessing potential physiological activities such as antioxidant and anti-inflammatory ones. Given the notable bioactivities of malvidin and its glycosides, there is significant promise for their utilization as beneficial dietary supplements. Nevertheless, limited research has specifically examined the potential applications of isolated or purified malvidin and its glycosides. Instead, the majority of studies has focused on investigating the properties of malvidin and its glycosides within plants or derived products. Cristian Del Bo′ et al. has suggested that anthocyanin-rich foods may improve cell antioxidant defense against DNA damage. In one study, ten young volunteers received one portion of blueberries or one portion of a control jelly. One portion (300 g) of the blueberries provided about 27 g of sugars (fructose and glucose) and 348 mg of anthocyanins (malvidin-3-galactoside, delphinidin-3-galactoside, and malvidin-3-arabinoside, making up more than 50% of the total anthocyanins content) [179]. The study conducted by Garcia-Alonso et al. explored the viability of employing an anthocyanidin-rich extract derived from *Vitis vinifera* grape peel. In the test meal, the consumed extract amount of 12 g contained a total of 183.8 mg of anthocyanin monoglucosides, with malvidin-3-glucoside being the most prevalent anthocyanin. The study aimed to evaluate the impact of red-wine-extract consumption on plasma antioxidant status and the production of monocyte chemoattractant protein 1 (MCP-1) in healthy individuals. Blood and urine samples were obtained from seven volunteers after the administration of the anthocyanin extract. The intact form of anthocyanins was detected in both plasma and urine samples, alongside other metabolites of anthocyanins. Furthermore, an enhancement in antioxidant capacity and a reduction in circulating levels of MCP-1 were observed in plasma [180]. Bakuradze conducted a 9-week study with 57 healthy male volunteers to investigate the biological effects of anthocyanin-rich fruit juice. The study included an initial 1-week washout period, followed by an 8-week intervention period where participants consumed either anthocyanin-rich fruit juice or a placebo. The red fruit juice had a total anthocyanin content of 274 mg/L, and among the detected anthocyanins were malvidin-3-glucoside and malvidin-3-galactoside. The anthocyanin-rich fruit juice demonstrated DNA-protective and antioxidant effects, leading to a significant reduction in background and total DNA strand breaks. Consumption of the anthocyanin-rich fruit juice also resulted in a significant decrease in body fat and an increase in fat-free mass. Furthermore, the activity of SOD was significantly elevated after consuming the anthocyanin-rich fruit juice, and the tested group showed decreased levels of LDL and total cholesterol. In conclusion, anthocyanin-rich fruit juice has the potential to improve DNA integrity and may have an impact on lipid metabolism in humans [181]. According to the studies of Mojica et al., black bean anthocyanin-rich extracts that contain malvidin-3-glucoside, via their ability to inhibit α-glucosidase, α-amylase, dipeptidyl peptidase-IV, and ROS and decrease glucose uptake, may have antidiabetic potential [178]. Kuntz et al. conducted a study to examine the effects of consuming beverages rich in anthocyanins on oxidation-related parameters in thirty healthy female volunteers. The participants were given 330 mL of beverages daily for 14 days, including a placebo, juice, and smoothie containing 8.9, 983.7, and 840.9 mg/L of anthocyanins, respectively. The juice and smoothie were made from a mixture of red grapes and bilberries in an 80:20 ratio. The main anthocyanin present in the juice and smoothie was malvidin-3-glucoside, with concentrations of 273 and 274 mg/mL, respectively. Blood and urine samples were collected before and after each intervention. Following the ingestion of anthocyanin-rich beverages, the subjects experienced a significant increase in plasma SOD and CAT activities, indicating enhanced antioxidant defense. The antioxidant capacity was also observed after consuming the juice and smoothie. Moreover, the concentrations of malondialdehyde, a marker of oxidative stress, decreased in both plasma and urine after consuming the anthocyanin-rich beverages. These findings suggest that anthocyanin-rich beverages may protect the body against oxidative stress, which is a key factor in the development of atherosclerosis [182].

Furthermore, several studies have investigated the effects of anthocyanins, including malvidin and its glycosides, on the human intestinal microbiota. Hidalgo et al. have suggested that malvidin-3-glucoside and its metabolites have the potential to influence the composition of the intestinal bacterial population by promoting the growth of *Bifidobacterium* spp. and *Lactobacillus–Enterococcus* spp. This positive modulation of the intestinal bacterial population is observed [183]. Vendrame et al. conducted a study on human volunteers to investigate the impact of consuming a wild blueberry drink for six weeks on the modulation of the intestinal microbiota. Each serving of the wild blueberry drink contained 375 mg of anthocyanins, including 49.5 mg of malvidin-galactose. The relative abundance of *Bifidobacterium* spp. significantly increased after the blueberry treatment, while *Lactobacillus acidophilus* levels also increased following the treatment. These findings suggest that regular consumption of a wild blueberry drink has a positive effect on the composition of the intestinal microbiota [184]. Zhou et al. conducted a study to assess the influence of blueberry anthocyanins on the human intestinal microbiota. Malvidin-3-glucoside was identified as the primary anthocyanin species, followed by malvidin-3-galactoside. The presence of anthocyanins was found to enhance the relative abundances of specific microbial communities, notably *Bifidobacterium* spp. These findings indicate that anthocyanins can significantly impact microbial diversity within the gut [185].

The multifaceted benefits of malvidin and its glycosides highlight their potential as nutrition enhancers. Not only can they provide appealing colors to food products, but they may also contribute to overall health and well-being. These findings suggest a potential market role for malvidin and its glycosides as functional food ingredients, offering both aesthetic and nutritional advantages. Further research and exploration of the properties and applications of malvidin and its glycosides are crucial in uncovering their full potential and maximizing their benefits for both the food industry and consumer health.

## 8. Conclusions

It can be concluded that malvidin and its glycosides possess significant anticancer, cardioprotective, antidiabetic, and neuroprotective properties due to their antioxidant and anti-inflammatory mechanisms of action. Various studies, including in vitro and in vivo, suggest that these molecules have the potential to counteract the onset and progression of several disease pathologies, in particular, with pathogenesis related to oxidative stress. Therefore, besides their colorant capacity, malvidin and its glycosides may have a wide range of health-promoting properties. However, further research is needed to fully understand the molecular mechanisms responsible for these effects and to explore potential new applications for these compounds. In addition, more detailed assessments of the efficacy of anthocyanin-rich products are required to support the development of new functional foods, dietary supplements, and pharmaceuticals. Overall, continued investigation into the biological activities of anthocyanins is essential for the development of novel therapeutic approaches and the promotion of public health.

## Figures and Tables

**Table 1:** Types of anthocyanins in fruits, vegetables, and their derivatives, with special emphasis in malvidin and its glycosides.

Source	Dominant Anthocyanins	Concentration	Refs.
Berry (*Berberis lycium* Royle)	Cyanidin-3,5-dihexoside, cyanidin-3-galatoside, cyanidin-3-glucoside, cyanidin-3-lathyroside, cyanidin-3-rut, delphinidin-3-glucoside, malvidin-3,5-dihexoside, pelargonidin-3,5-diglucoside, pelargonidin-3-pentoxilhexoside, pelargonidin-3-rutinoside, pelargonidin-hexoside, peonidin-3-rutinoside	Malvidin-3,5-dihexoside: 4.21%	[36]
Bilberry (*Vaccinium myrtillus* L.)	Cyanidin-3-arabinoside, cyanidin-3-galatoside, cyanidin-3-glucoside, delphinidin-3-arabinoside, delphinidin-3-glucoside, delphinidin-3-galatoside, malvidin-3-arabinoside, malvidin-3-galatoside, malvidin-3-glucoside, peonidin-3-arabinoside, peonidin-3-galatoside, peonidin-3-glucoside, petunidin-3-arabinoside, petunidin-3-galatoside, petunidin-3-glucoside	Malvidin-3-arabinoside: 175–295 mg/100 g dry weight; malvidin-3-galatoside: 82–127 mg/100 g dry weight; malvidin-3-glucoside: 344–506 mg/100 g dry weight	[37]
Blackberry (*Rubus fruticosus* L.)	Cyanidin, cyanidin-3-glucoside, cyanidin-3-arabinoside, malvidin-3-galactoside, malvidin-3-glucoside	-	[38,39]
Blueberry (*Vaccinium corymbosum* L.)	Cyanidin-3-arabinoside, cyanidin-3-galatoside, cyanidin-3-glu, delphinidin-3-arabinoside, delphinidin-3-galatoside, delphinidin-3-glucoside, malvidin-3-arabinoside, malvidin-3-galatoside, malvidin-3-glucoside, peonidin-3-galatoside, peonidin-3-glucoside, petunidin-3-arabinoside, petunidin-3-galatoside, petunidin-3-glucoside	Malvidin-3-arabinoside: 147.6–697 mg/100 g dry weight Malvidin-3-galatoside: 137–330 mg/100 g dry weight Malvidin-3-glucoside: 3–200.4 mg/100 g dry weight	[37,40,41]
Dabai (*Canarium odonthophyllum* Miq.)	Delphinidin, cyanidin, cyanidin-3-glucoside, cyanidin-3-rutinoside, malvidin-3,5-diglucoside, pelargonidin, peonidin-3-glucoside	Malvidin-3,5-diglucoside: 0.07–0.20 mg/100 g dry weight	[42]
Red grape (*Vitis vinifera* L.)	Cyanidin-3-glucoside, delphinidin-3-glucoside, malvidin-3-acetylglucoside, malvidin-3-glucoside, malvidin-3-*p*-coumarylglucoside, peonidin-3-acetylglu, peonidin-3-glucoside, peonidin-3-*p*-coumarylglucoside, petunidin-3-glucoside	Malvidin-3-acetylglucoside: 1.51–29.22% Malvidin-3-glucoside: 32.40–58.96% Malvidin-3-*p*-coumarylglucoside: 7.29–18.46%	[43]
Red wine	Cyanidin-3-glucoside, delphinidin 3-glucoside, malvidin-3-acetylglucoside, malvidin-3-coumarylglucoside, malvidin 3-glucoside, peonidin-3-acetylglucoside, peonidin 3-glucoside, peonidin-3-ρ-coumarylglucoside, petunidin 3-glucoside	Malvidin-3-acetylglucoside: 2.42–144.27 mg/L Malvidin-3-coumarylglucoside: 2.48–19.96 mg/L Malvidin 3-glucoside: 125.44–353.13 mg/L	[44,45]
Sweet cherries (*Prunus avium* L.)	Cyanidin 3-rutinoside, cyanidin-3-5-diglucoside, cyanidin-3-arabinoside, cyanidin-3-coumaroyl-diglucoside, cyanidin-3-glucoside, cyanidin-3-glucoside, cyanidin-3-rutinoside, cyanidin-3-rutinoside, cyanidin-3-sambubioside, cyanidin-3-sophoroside, delphinidin 3-rutinoside, malvidin-3-glucoside-acetaldehyde, pelargonidin 3-rutinoside, pelargonidin-3-glucoside, pelargonidin-3-rutinoside, pelargonidin-3-rutinoside, peonidin 3-rutinoside, peonidin-3-glucoside, peonidin-3-rutinoside, peonidin-3-rutinoside	Malvidin-3-glucoside-acetaldehyde: 0.08–011 mg/100 g fresh weight	[46,47,48]
Tomato (*Solanum lycopersicum* L.)	Delphinidin-glycoside, delphinidin-rutinoside, malvidin 3-glucoside, malvidin-glycoside, malvidin-ρ-coumaroyl-rutinoside-glycoside, petunidin rutinoside, petunidin ρ-coumaroyl-rutinoside, petunidin ρ-coumaroyl-rutinoside-glycoside	Malvidin 3-glucoside: 54.77–298.57 μg/1 g dry weight	[49,50]

**Table 2:** Antioxidant activities of malvidin and its glycosides evaluated in an in vitro model.

Compounds	Cell Line	Effect	Refs.
Malvidin Malvidin-3-glucoside Malvidin-3-galactoside	Human umbilical vein endothelial cells	Decreased: ROS levels and xanthine oxidase enzyme activity Increased: superoxide dismutase enzyme activity	[59]
Malvidin 3,5-diglucoside	Human endothelial cells	Decreased: ROS levels	[60]
Malvidin-3-glucoside	Bovine aortic endothelial cells pretreated with peroxynitrite	Decreased: ROS levels Inhibited mitochondrial apoptotic signaling pathways by preventing mitochondrial membrane depolarization, activation of caspase-3 and -9, and reducing the expression of the proapoptotic Bax protein	[61]
Malvidin-3-glucoside, Malvidin-3-galactoside	Human retinal pigment epithelial cells pretreated with H_2_O_2_	Decreased: ROS and malondialdehyde levels Increased: superoxide dismutase, catalase, and glutathione peroxidase enzymes activity	[62]
Malvidin-3-arabinoside	Human colorectal adenocarcinoma cells pretreated with ethyl carbamate	Decreased: oxidative damages Enhancing autophagy flux	[63]
Malvidin	human fibroblast cells (WI-38) pretreated with H_2_O_2_	Decreased: lipid peroxidation	[64]

**Table 3:** Anti-inflammatory activities of malvidin and its glycosides evaluated in an in vitro model.

Compounds	Cell Line	Effect	Refs.
Malvidin	Human umbilical vein endothelial cells pretreated with tumor necrosis factor-alpha	Decreased: monocyte chemotactic protein-1, intercellular adhesion molecule-1, and vascular cell adhesion molecule-1 production Inhibition: degradation of IκBα and the nuclear translocation of p65	[72]
Malvidin, malvidin-3- glucoside, malvidin-3-galactoside	Human umbilical vein endothelial cells pretreated with tumor necrosis factor-alpha	Decreased: monocyte chemotactic protein1, intercellular adhesion molecule-1, vascular cell adhesion molecule-1 production and angiotensin I-converting enzyme activity Inhibition: degradation of IκBα and the nuclear translocation of p65	[73]
Malvidin-3-glucoside and malvidin-3-galactoside	Human umbilical vein endothelial cells pretreated with tumor necrosis factor-alpha	Decreased: monocyte chemo-tactic protein-1, intercellular adhesion molecule-1, and vascular cell adhesion molecule-1 production Inhibition: degradation of IκBα and the nuclear translocation of p65	[74]
Malvidin-3-glucoside	Bovine arterial endothelial cells pretreated with peroxynitrite	Decreased: inducible nitric oxide synthase activity, cyclooxygenase activity, IL-6 production Increased: eNOS activity and NO production	[75]
Malvidin	RAW264.7 macrophages stimulated by bacterial lipopolysaccharide	Decreased: lipopolysaccharide-induced nuclear factor-kappaB, poly ADP-ribose polymerase and mitogen-activated protein kinase activation, reactive oxygen species production and mitochondrial depolarization Increased: mitogen-activated protein kinase phosphatase-1 expression and Akt activation	[76]
Malvidin-3-glucoside	Rat macrophages stimulated by bacterial lipopolysaccharide	Decreased: tumor necrosis factor-alpha, IL-1, IL-6 and inducible nitric oxide synthase activity	[77]
Malvidin	Human monocytic cells (THP1) stimulated by bacterial lipopolysaccharide	Decreased: IL-6, tumor necrosis factor-α, and IL-1β production Increased: IL-10 production	[78]
Malvidin	Peripheral blood mononuclear cells stimulated by bacterial lipopolysaccharide	Decreased: IL-6, tumor necrosis factor-alpha and IL-1β production, cyclooxygenase 2 activity	[79]
malvidin	Human fibroblast cells (WI-38) pretreated with H_2_O_2_	Decreased: NF-κB production and cyclooxygenase 2 and inducible nitric oxide synthase activity	[64]

## Data Availability

Not applicable.
